# Inhibition of Enveloped Virus Surrogate Phi6 Infection Using Yeast-Derived Vacuoles

**DOI:** 10.1128/spectrum.02661-22

**Published:** 2023-01-23

**Authors:** Wooil Choi, Yang-Hoon Kim, Jiho Min

**Affiliations:** a Graduate School of Semiconductor and Chemical Engineering, Jeonbuk National University, Jeonbuk, South Korea; b School of Biological Sciences, Chungbuk National University, Cheongju, South Korea; University of Georgia

**Keywords:** yeast-derived vacuole, antiviral agents, enveloped virus, enzyme bomb, infection inhibition

## Abstract

The periodic emergence of infectious disease poses a serious threat to human life. Among the causative agents, including pathogenic bacteria and fungi, enveloped viruses have caused global pandemics. In the last 10 years, outbreaks of severe acute respiratory syndrome coronavirus 2 disease, severe acute respiratory syndrome, and Middle East respiratory syndrome have all been caused by enveloped viruses. Among several paths of secondary transmission, inhalation of aerosols containing saliva with sputum droplets from infected patients is the major path. To prevent these infectious diseases, mass use of antiviral agents is essential. The yeast-derived vacuole is a small organelle in which hydrolytic enzymes are concentrated. It is an intracellular organ with an excellent ability to process old organelles and bacteria and viruses that have invaded from the outside and can be present in sufficient quantity to be called a kind of enzyme bomb. We confirmed the inhibition of virus infection and structural collapse by vacuole treatment. Among several enzymes, proteases affected Phi6 infectivity. This study tried to isolate these vacuoles from yeast and use them as an antiviral agent for virus treatment, which is a recent issue. We confirmed that viral infectivity was inactivated, and structure collapsed through vacuole treatment. This paper is meaningful in that extracellularly isolated yeast-derived vacuoles are a first attempt to utilize vacuoles for viral treatment.

**IMPORTANCE** The study assesses the vacuoles isolated from the yeast Saccharomyces cerevisiae as green antiviral agents to decrease the concerns about massive use of chemical antiviral agents and its side effects. To prevent the spreading of infectious diseases, personal or public use of antiviral agents is encouraged. The concern about the active compounds of these chemical antiviral agents has grown. Active compounds of antiviral agents have potential side effects on human health and the environment. Our proposed approach suggests effective and green antivirus material from a nonhazardous yeast strain. Also, large-scale production using a fermentation process can allow cost-effectiveness. The results showed sufficient reduced infectivity by vacuole treatment. The exposed vacuole can play the roles of both enzyme bomb to the virus and renewable nutrient source in the ecosystem.

## INTRODUCTION

The outbreak of infectious diseases caused by enveloped viruses, such as the zika virus, influenza virus, hepatitis B virus, hepatitis C virus, the coronavirus responsible for severe acute respiratory syndrome (SARS), and the virus responsible for Middle East respiratory syndrome, poses a serious threat to human health (1). In particular, the global pandemic of SARS coronavirus disease in 2019 (SARS-CoV-2) has adversely affected both human health and life.

Enveloped viruses infect host cells by inducing the fusion of the viral envelope with cell membrane. The envelopes are normally derived from the membranes of host cells and can help the virus avoid the host immune system. Spike proteins on the surface of the envelope can bind to the target host, and fusion of the virus with host cells can be accomplished. In this regard, structural collapse of the virus can be one strategy to prevent infection with the enveloped virus (1).

Bacteriophage Phi6 is widely used as a surrogate for enveloped viruses and offers the advantage of lab safety. Phi6 is nonpathogenic to humans and requires World Health Organization biosafety level 1 control measures. Phi6, a kind of lytic virus phage, is a member of the *Cystoviridae* and has a double-stranded RNA (dsRNA) genome of 13.4 kbp with three segments (L, M, and S), a diameter of 75 nm, a nucleoprotein capsid diameter of 60 nm, and 10 major coat proteins (2). Several research reports have assessed the suitability of Phi6 as a surrogate for environmental persistence related to enveloped viruses, including Ebola virus and coronavirus (3, 4).

Considering the rate of virus spread and infection, the use of antiviral agents is very important. Among several paths of secondary transmission, inhalation of aerosols containing saliva with sputum droplets from infected patients is the major path (5). To prevent these infectious diseases, mass use of antiviral agents should be essential. Alcohol-, chlorine-, or quaternary ammonium compound-based antiviral agents can damage the virus and decrease its infectivity. These chemical antiviral agents are considered economical. However, there are concerns that massive environmental exposure to antiviral agents can adversely affect the ecosystem. Although a few areas of cities can avoid contact with these chemicals, almost all organisms are directly or indirectly exposed to them. Also, while massive use of a chemical antiviral agent has not yet been proven effective, its harmful effects on the environment are certain (6). For example, chlorine antiviral agents in wastewater react with organic matter in the environment and create organic chlorine compounds that can be toxic and persistent environmental contaminants (7). For both this pandemic state and over the long term, green materials that provide alternatives to toxic antiviral agents must be explored.

The typical eukaryote has a lysosome or vacuole that is a digestive organelle within the cell. It contains acidic hydrolase to degrade and digest the intra- and extracellular wastes. Vacuolar enzymes, including proteases, glycosidases, phosphohydrolases, and lipases, maintain physiological homeostasis by recycling nutrients (8, 9). Invading bacteria or viruses can be degraded by vacuolar enzymes. For this reason, the isolated vacuole from budding yeast has antibacterial and anticancer effects (10).

In this study, to decrease the concern about massive use of chemical antiviral agents and their side effects, we assessed the vacuoles isolated from the yeast Saccharomyces cerevisiae as a green antiviral agent. To investigate decreased infectivity by vacuole treatment, a plaque assay was conducted. Structural disruption of bacteriophage Phi6 was confirmed by reverse transcription-quantitative PCR (RT-qPCR) and transmission electron microscopy (TEM) imaging (11, 12).

## RESULTS

### Effect of vacuole treatment on infectivity of the enveloped virus surrogate Phi6.

**FIG 1:**
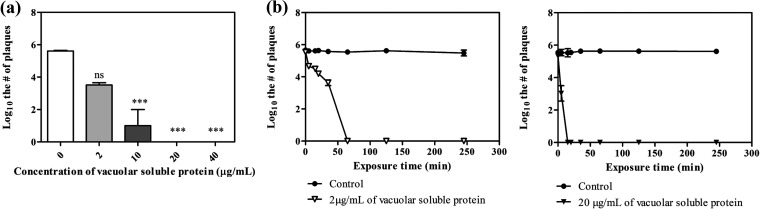
Titers of Phi6 versus vacuole concentration (*P* < 0.0001) (a) and versus exposure time (*P* = 0.0003 and 0.0002, respectively) (b).

To assess the antiviral activity of isolated vacuoles from yeast, 5 × 10^5^ PFU/mL of Phi6 was treated with vacuoles at concentrations of 1, 5, 10, and 20 μg/mL of soluble protein-containing vacuoles for 30 min, and 2 and 20 μg/mL of soluble protein-containing vacuole was treated for each exposure time, 5, 10, 20, 35, 65, 125, and 245 min, and confirmed by plaque assay. The vacuole treatment was conducted at 25°C. After titer determinations for treated Phi6, the results shown in Fig. 1 confirmed the decrease of infectivity. Fig. 1a and b show that infectivity decreased, with dependence on soluble protein concentration and exposure time. Table 1 shows that when we treated 2 or 20 μg of soluble protein-containing vacuoles for 60 or 5 min, respectively, we could confirm the reduced infectivity (>99.99%).

**TABLE 1:** Antivirus activity of vacuole treatment against Pseudomonas Phi6

Concn of vacuolar soluble protein (μg/mL)	Exposure time (min)	Log(PFU) (±SD)		Relative infectivity (% reduction)
		Negative control	Treatment	
2	0	5.66 (±0.05)	5.54 (±0.03)	2.12
	5	5.61 (±0.05)	4.66 (±0.09)	88.69
	10	5.62 (±0.07)	4.50 (±0.21)	91.37
	20	5.64 (±0.01)	4.20 (±0.17)	95.62
	35	5.58 (±0.11)	3.63 (±0.25)	98.79
	65	5.55 (±0.15)	<0	≥99.99
	125	5.62 (±0.07)	<0	≥99.99
	245	5.49 (±0.26)	<0	≥99.99
20	0	5.55 (±0.04)	5.31 (±0.12)	4.32
	5	5.54 (±0.16)	3.03 (±0.39)	99.84
	10	5.53 (±0.39)	<0	≥99.99
	20	5.55 (±0.09)	<0	≥99.99
	35	5.63 (±0.07)	<0	≥99.99
	65	5.63 (±0.05)	<0	≥99.99
	125	5.63 (±0.07)	<0	≥99.99
	245	5.62 (±0.05)	<0	≥99.99

### Degradation of enveloped virus by isolated vacuoles from yeast.

**FIG 2:**
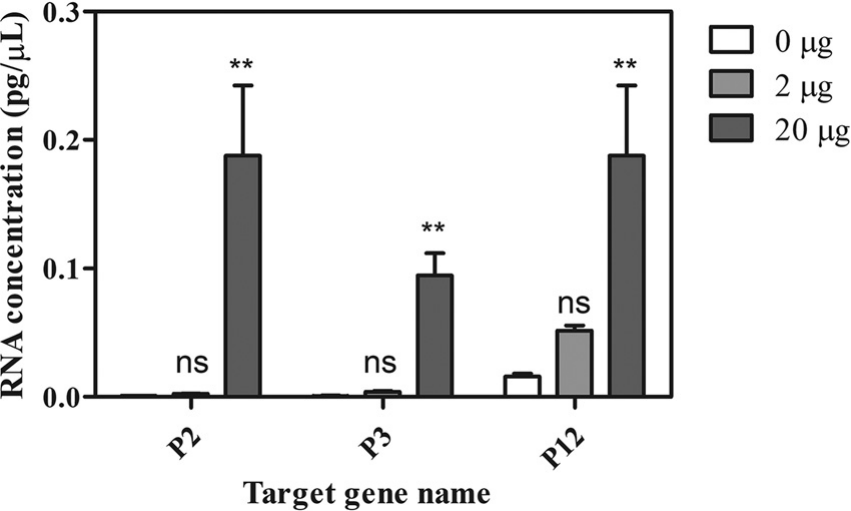
Amplification and quantification of leaked RNA by RT-qPCR after vacuole treatment (*P* < 0.005).

To investigate the structural collapse of virus envelope and capsid, leaked dsRNA following the degradation of structures was amplified by RT-qPCR. The biggest parts (P2, P3, and P12) of each segment (L, M, and S) were chosen, respectively. After treatment of vacuoles, the supernatant after centrifugation at 20,000 × *g* was used as RNA template. When we compared the amount of amplified RNA, the concentration of RNA amplicons was increased. Fig. 2 shows that a maximum of 11-fold leaked RNA was observed.

### Morphology of virus after vacuole treatment.

**FIG 3:**
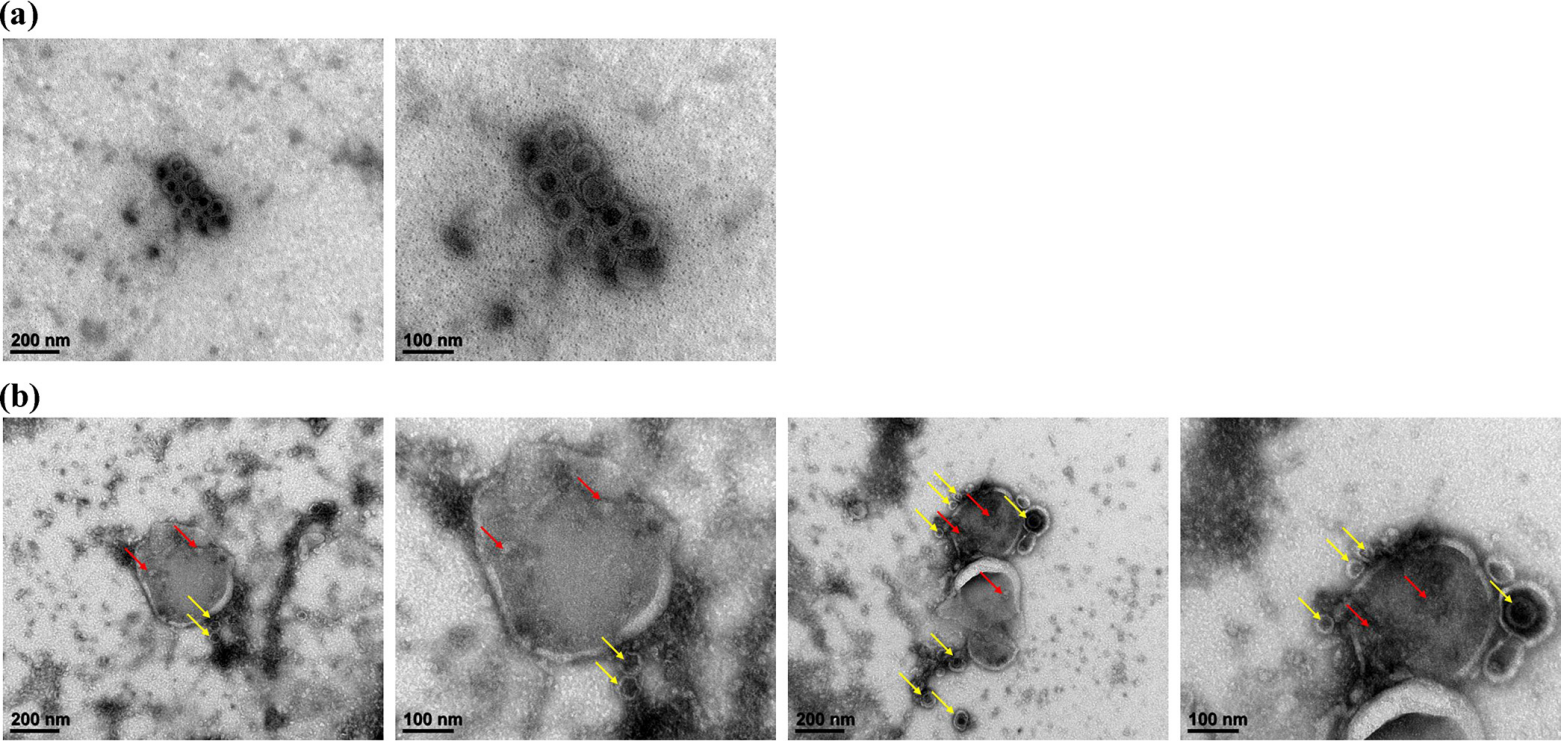
Morphology investigation of Phi6 and vacuoles using TEM images. (a) Bacteriophage Phi6; (b) Phi6 treated with vacuole.

The TEM images of Phi6 and vacuoles in Fig. 3a and b show Phi6 particle sizes and structural collapse. In Fig. 3a, it can be confirmed that the size of Phi6 viruses was maintained consistently around 70 ± 2 nm, and the double-layer enveloped and icosahedral structures were well maintained. However, in the case of treatment with vacuoles (Fig. 3b), it was confirmed that some virions were observed in the vacuolar lumen (indicated by the red arrows), and some Phi6 were observed to be spherical rather than icosahedral with envelopes removed (indicated by the yellow arrows in Fig. 3b). Therefore, treatment with vacuoles resulted in a morphological change of the virions which was considered to show inhibition of viral infection.

### Antivirus activities of protease and lipase.

Yeast vacuoles have several hydrolytic enzymes. Among the enzymes, proteases and lipases can affect antiviral activity. After treatment with protease or lipase from pancreas, the relative infectivity reduction was determined. When 10 U/mL of protease was added, the relative infectivity reduction was over 99.99%; however, in the case of lipase, there was not sufficient antivirus effect (Table 2).

**TABLE 2:** Antivirus activity of enzyme treatment against Pseudomonas Phi6

Enzyme (at 10 U/mL)	Temp (°C)	Log(PFU) (±SD)		Relative infectivity (% reduction)^*a*^
		Negative control	Treatment	
Protease	25	5.50 (±0.01)	4.32 (±0.06)	93.42*
	37	5.45 (±0.01)	<0	≥99.99*
Lipase	25	5.50 (±0.01)	5.63 (±0.02)	−33.23
	37	5.45 (±0.01)	5.54 (±0.03)	−21.63

a *, *P* < 0.0001 (significantly different from control group).

## DISCUSSION

To prevent the spread of infectious diseases, personal or public use of antiviral agents is encouraged. In the global pandemic caused by SARS-CoV-2, an enveloped virus, the practice of chemical disinfection was intensified. Nowadays, concern about the active compounds of these chemical antiviral agents has grown. Interaction between the active compounds of antiviral agents has potential side effects on human health and the environment (13). Here, we evaluated yeast-derived vacuoles as alternative antiviral agents for sustainability.

Yeast vacuoles contain 360 polypeptides in the soluble fraction in the lumen. Most of the identified proteins are canonical vacuolar proteases, glycosidases, phosphohydrolases, lipid-binding proteins, or nonspecific fractions. Among several enzymes, seven vacuolar proteases have been characterized, including three serine proteases, one aspartyl protease, and three metalloproteases (8, 9). Serine proteases can degrade the surface of bacteria and decrease bacterial viability when exposed to the host immune system, and they are applied as antibacterial materials (14, 15). The components of the enveloped virus include spike protein, lipid envelope, protein matrix, and nucleocapsid. Proteins account for most of the viral survival and infectivity. According to our results, isolated vacuoles from yeast can reduce the infectivity of Phi6 and affect the virus structure. In particular, some proteases in vacuoles were active antiviral compounds in this study. In humans, enveloped virus enters the host cell through receptor-mediated endocytosis, and uncoating of envelope is carried out by lysosomes. The activation of endolysosomal proteases, controlled by ion exchange, is required for cleavage of the viral S glycoprotein (16). The entrance and exposure of viruses into vacuoles can be assumed by membrane composition and infection mechanism of enveloped viruses. Vacuolar membranes contain phosphatidylcholine (the most abundant, 46.5%), followed by phosphatidylethanolamine, phosphatidylinositol, and phosphatidylserine (17). Enveloped virus can be fusion with theses lipid composition like infection into host cells (18). In case of mycoviruses, they are considered to lack infectivity through extracellular transmission, because rigid cell walls of fungi can be strong barriers against the uptake of virus; in some reports, however, protoplasts of fungi have been infected by virus particles (19). In this study, the rigid compartment of yeast cell walls was removed, and vacuoles were isolated. Combining the structural similarities and mechanism of viral infection, these antiviral mechanisms can be initiated (20, 21). After viral entrance, the digestive system of the cell helps the virus copy itself. However, uncoating the viral protein before infection can block the access into the host cell. The TEM images confirmed the damage of envelope and matrix. Also, leaked dsRNA was amplified by reverse transcription-PCR. These results demonstrated the antivirus effect of isolated vacuoles. Also, degradation of leaked RNA can be expected by RNases such as RNase T_2_-like. RNase T_2_-like is released from the vacuole to the outside during stress conditions. Thus, released RNA can be degraded and does not affect secondary transmission. When Phi6 was exposed to vacuoles for a long time, leaked RNA was not amplified by RT-qPCR (data not shown) (22).

In conclusion, the use of antiviral agents has been essential to protect human health from the continued emergence of infectious disease. If the emergence of virus cannot be prevented, the properties of antiviral agents must be changed for environmental sustainability. In this study, the proposed approach demonstrated effective and green antivirus material from a nonhazardous yeast strain. Also, large-scale production of this material using the fermentation process could allow cost-effectiveness. The results showed sufficient reduced infectivity by vacuole treatment. The exposed vacuole can play the roles of both enzyme bomb to the virus and renewable nutrient source in the ecosystem. In the current study, yeast-derived vacuoles were suggested as an alternative to chemical antiviral agents that cause environmental pollution. Through this research, we can show that a biological approach to prevent infectious disease can be effective and suitable for our system.

## MATERIALS AND METHODS

### Preparation of bacteriophage Phi6 and host bacteria.

Bacteriophage Phi6 (Pseudomonas phage Phi6; DSM 21518) and its host, Pseudomonas syringae (Pseudomonas sp. HER1102, DSM 21482), were purchased from DSMZ, Germany. Pseudomonas syringae was propagated in tryptic soy broth (TSB; Beckton Dickinson [BD]), and Phi6 was maintained in SM buffer (0.1 M NaCl, 8 mM MgSO_4_, 50 mM Tris-HCl; pH 7.5). Phi6 was grown in host cells by a two-layer method. Briefly, host bacteria were cultured at 26°C in a shaking incubator until the optical density at 600 nm (OD_600_) reached 0.8 and inoculated with Phi6 stock solution at a 1:1 ratio. A mixed solution of host and virus was put in 5 mL of TSB-top agar (0.75% agar in TSB) and poured on the TSB-bottom agar (1.5% agar in TSB). After pouring the top agar, the inoculated TSB plate was incubated at 26°C overnight. The TSB-top agar was harvested by cell scraper and eluted in SM buffer for 90 min. After elution, the TSB-top agar was discarded by centrifugation at 2,200 × *g* for 10 min. The residue was then eliminated by centrifuge at 9,800 × *g* for 5 min. The collected supernatant was mixed with 8% PEG–0.5 M NaCl and put on ice for 60 min. Supernatant was discarded by centrifugation at 4°C, 10,000 × *g*, for 15 min, and pellet was resuspended in SM buffer. Finally, the precipitated Phi6 was purified by 0.2-μm-pore-size syringe filter. Phi6 and P. syringae were stored at −70°C in 10% (vol/vol) glycerol in SM buffer and TSB, respectively. Table 3 lists the bacteria and virus strains used in this study.

**TABLE 3:** Strain information

Organism	Strain designation	Reference strain
Saccharomyces cerevisiae	BJ3501	ATCC 208280
Pseudomonas syringae	HER1102	DSM 21482
Pseudomonas phage Phi6	HER102	DSM 21518

### Isolation of vacuoles from S. cerevisiae.

To isolate vacuoles from S. cerevisiae (ATCC 208280), yeast was cultured with 2 steps at 30°C in a shaking incubator for 24 h (10). After seed culture, the OD_600_ of the culture solution was 0.6. The main culture was conducted with 1% (vol/vol) of seed solution. The yield of harvested cells was approximately 10 g/liter. Yeast culture was followed by vacuole isolation. Briefly, 5 g of yeast cells was resuspended with Tris-SO_4_ buffer (100 mM Tris-SO_4_ [pH 9.4], 10 mM dithiothreitol [DTT]) and incubated at 30°C in a shaking incubator for 15 min. After making the cell wall mushy, it was centrifuged at 2,200 × *g* for 10 min, and supernatant was discarded. Glass beads were added to cells in a mass ratio of 1:1 and resuspended in breaking buffer (20 mM Tris-HCl [pH 7.4], 600 mM sorbitol, and 1 mM phenylmethylsulfonyl fluoride [PMSF]). Mixtures were repeatedly vortexed and kept on ice at an interval of 1 min for a total of 20 min. After 5 min of stabilization on ice, centrifugation was carried out at 500 × *g* for 5 min. Supernatant was collected into a microtube and then centrifuged at 20,000 × *g* for 30 min at 4°C (10, 23).

To quantify the isolated vacuoles, vacuolar soluble protein was measured. The obtained vacuoles were vortexed 10 times (1 min on and off) with lysis buffer (0.1% NP-40, 5 mM DTT, and 0.1 mM PMSF) at a ratio of 1:1 and allowed to react on ice for 30 min. After stabilization on ice, centrifugation was carried out at 16,000 × *g*, and supernatant was measured in a Bradford assay (Bio-Rad, Richmond, Ca, USA).

### Nanoparticle tracking analysis.

To validate the size distribution of vacuoles, nanoparticle tracking analysis was performed. The vacuoles were diluted 1:20,000, and the diluted vacuole solutions were injected into a Zetaview system (Particle Metrix GmbH, Germany).

### Treatment of yeast vacuoles with different concentrations and time.

To confirm the decrease of Phi6 infectivity, isolated yeast vacuoles were treated with different concentrations and exposure times. Phi6 at 5 × 10^5^ PFU/mL was treated with each concentration (1, 5, 10, and 20 μg/mL) of soluble protein-contained vacuole for 35 min at 25°C, and 2 or 20 μg of soluble protein-contained vacuole was treated for each exposure time (5, 10, 20, 35, 65, 125, and 245 min) at 25°C, respectively. SM buffer was used as a negative control and also for virus dilution. After exposure, vacuoles were discarded by centrifugation at 20,000 × *g*.

### Plaque assay.

After vacuole treatment, vacuoles were separated using centrifugation at 16,000 × *g*, and pellet was discarded. A plaque assay was carried out to confirm the decrease of infectivity. Each sample was prepared by 10-fold serial dilution using SM buffer and used to infect to P. syringae. The infected samples were transferred into the soft TSB-top agar and immediately overlaid onto the TSB-bottom agar plate. After pouring the top agar, the inoculated TSB plate was incubated at 26°C overnight, and the number of plaques was counted.

### Degradation of virus structure, RNA isolation, amplification, and quantification.

To investigate the leaked RNA and structural collapse of the enveloped virus, RT-qPCR was carried out. After treatment of vacuoles for 10 min, centrifugation at 16,000 × *g* was carried out for separation of vacuoles and supernatant as RNA template. Representative parts (P2, P3, and P12) of each segment (L, M, and S, respectively) were amplified and detected. A total of 2 × 10^5^ copies/μL of transcribed poly(A)^+^ RNA was used as a positive control RNA for RNA quantification using an RNA PCR kit (AMV; version 3.0; TaKaRa, Japan). RT-qPCR experiments were carried out using a real-time PCR detection system (CFX connect RT-PCR detection system, Bio-Rad Laboratories, Inc., USA) and one-step RT-qPCR (AccuPower GreenStar RT-qPCR, Bioneer Co., South Korea). The probe used in RT-qPCR was SYBR green I for detection and quantification of amplicon. PCR products were analyzed using CFX Maestro software (Bio-Rad Laboratories, Inc., USA). The primer pair used in the PCR experiment was designed with reference to the sequence obtained from NCBI. The gene IDs of the sequences were as follows: P2 (NCBI gene ID 956436), P3 (NCBI gene ID 956441), and P12 (NCBI gene ID 956431) (see Fig. S1 in the supplemental material). Primer pairs used for RT-qPCR were synthesized from AcuuOligo (Bioneer Co., South Korea) (Table 4).

**TABLE 4:** Primer pairs for RT-PCR

Target gene (segment portion)	Primer sequences	Gene position	Amplicon size (bp)
RNA-directed RNA polymerase (P2)	5′-AAATGCCGAGGAGAGCTCCCGCGTT-3′; 5′-CGCTTACCTCGGCATTACAGAACGGAG-3′	Segment L (nt 943–2940)	1,995
Spike protein (P3)	5′-ATGCGCTACCAAGGCATCAACGAGT-3′; 5′-CAGTCAGCAGCCATACGACTCCTTA-3′	Segment M (nt 1475–3411)	1,936
Morphogenetic protein (P12)	5′-CGCATGGTTATCGGTCTCCTGAAGT-3′; 5′-ACCTTACGGAACATCCTTACGGTGG-3′	Segment S (nt 754–1341)	585

### Transmission electron microscopy.

After vacuole isolation and treatment, the morphology of vacuoles and Phi6 was confirmed by TEM. Phi6 at 10^10^ PFU/mL was put on the grid. Bacteriophages were negatively stained with 1% uranyl acetate for a few seconds. TEM images were analyzed with an H-7650 system (Hitachi, Japan) installed in the Center for University-wide Research Facilities at Jeonbuk National University. Microscopy was operated at 100 kV, and images were acquired at magnifications of ×100,000 and ×200,000.

### Antivirus activities of protease and lipase.

To confirm which enzymes decreased the infectivity of the virus, protease and lipase were analyzed. Protease from bovine pancreas was purchased from Sigma-Aldrich, USA (catalog number P4630), while lipase was purchased from Abcam, United Kingdom (catalog number ab102524). Phi6 at 5 × 10^5^ PFU/mL was treated with enzyme at 10 U/mL for 10 min. Reduction of infectivity was confirmed by plaque assay.

### Statistical comparisons.

In this study, the differences between two groups of control and experimental groups were analyzed using Student's *t* test. One-way analysis of variance was used for comparisons of more than two groups, and multiple comparisons were performed using Tukey-Kramer *post hoc* test in GraphPad Prism 5. Each data point was obtained from more than three independent experiments conducted simultaneously for error analysis. Data are presented as means ± standard errors of the means (SEM). Error bar indicate SEM.

### Data availability.

We confirm that the data supporting the findings of this study are available within the article and its supplemental material.
