# Supplementary material for: Inhibition of Enveloped Virus Surrogate Phi6 Infection Using Yeast-Derived Vacuoles
Source: Microbiol Spectr. 2023 Jan 23;11(1):e02661-22. doi: 10.1128/spectrum.02661-22 (PMC9927162; doi:10.1128/spectrum.02661-22)
Supplement: Supplemental file 1 — Supplemental material. Download spectrum.02661-22-s0001.pdf, PDF file, 0.3 MB [file spectrum.02661-22-s0001.pdf]

Supplementary File for Review

FIG S1. Pseudomonas Phi6 gene information

RNA polymerase, P2 in Segment L, (NCIB gene ID: 956436)

https://www.ncbi.nlm.nih.gov/gene/?term=956436

ATGCCGAGGAGAGCTCCCGCGTTCCCTCTGAGCGATATCAAGGCTCAGATGCTGTTCGCAAATAACATCAAGGCCCAACAAGCCTC
GAAGCGTAGCTTCAAAGAGGGGGCGATTGAAACGTACGAAGGGCTGCTTTCAGTAGACCCTCGGTTTTTGAGTTTCAAGAACGAG
CTCTCTCGGTATCTGACCGACCACTTCCCGGCGAACGTCGACGAGTATGGTCGTGTTTATGGAAACGGTGTTCGTACCAACTTCTTT
GGTATGCGCCACATGAACGGGTTTCCAATGATCCCCGCGACGTGGCCACTCGCTTCCAACCTTAAGAAACGTGCCGACGCTGACCT
AGCCGATGGCCCTGTTTCTGAGCGCGACAATCTACTCTTTCGCGCCGCAGTCCGGCTTATGTTTTCAGATCTAGAGCCTGTTCCGCT
GAAGATCCGTAAAGGATCGTCAACCTGCATCCCGTATTTTTCTAACGATATGGGAACGAAGATCGAGATCGCCGAGCGCGCTCTTG
AGAAAGCGGAAGAAGCTGGCAATCTGATGCTGCAAGGTAAGTTTGATGACGCCTACCAGCTCCACCAAATGGGTGGTGCCTATTA
CGTCGTGTATCGTGCACAATCGACCGATGCTATCACACTCGACCCTAAGACCGGAAAATTCGTGTCAAAGGATCGTATGGTCGCTG
ACTTCGAATACGCAGTCACGGGCGGTGAGCAAGGCTCGCTGTTCGCTGCTTCGAAGGATGCCTCTCGTTTGAAGGAACAGTACGG
GATAGATGTCCCGGACGGGTTTTTCTGCGAGCGGCGTCGTACCGCTATGGGTGGTCCGTTCGCGTTGAACGCTCCTATCATGGCCGT
TGCGCAACCTGTGCGAAACAAAATTTACTCCAAGTACGCTTACACCTTTCACCATACTACTCGTCTTAATAAGGAGGAAAAGGTGA
AAGAGTGGTCGTTGTGCGTCGCTACTGACGTATCCGACCACGACACGTTCTGGCCTGGATGGCTGCGGGATCTCATCTGTGATGAA
CTGCTCAACATGGGGTACGCTCCGTGGTGGGTTAAGTTGTTCGAGACCTCGCTCAAACTGCCCGTTTACGTGGGCGCTCCTGCTCC
TGAGCAGGGCCACACGTTGTTGGGTGATCCGTCCAACCCTGATCTCGAAGTTGGTCTCTCGTCCGGACAAGGGGCGACCGACCTC
ATGGGCACGTTGCTCATGAGTATCACCTACCTGGTGATGCAACTTGATCACACCGCTCCTCACCTCAACAGTCGAATCAAGGACAT
GCCATCAGCATGCCGCTTTCTTGACTCGTATTGGCAAGGACACGAGGAGATCCGTCAGATCTCAAAATCTGATGATGCTATACTTGG
CTGGACCAAAGGTCGTGCTTTGGTTGGTGGTCATCGTTTGTTCGAGATGCTGAAAGAGGGTAAGGTTAACCCCTCACCTTACATGA
AGATCTCCTACGAGCACGGTGGCGCCTTCCTTGGTGACATCCTGCTTTACGACTCGCGTCGTGAGCCTGGCTCTGCCATCTTCGTTG
GTAACATCAACTCAATGCTGAACAACCAGTTCAGCCCTGAGTACGGTGTCCAATCGGGCGTTCGCGACCGATCTAAGCGCAAACG
GCCGTTCCCCGGTCTTGCTTGGGCGTCGATGAAAGATACCTACGGTGCCTGTCCGATCTACTCTGATGTGCTGGAGGCGATCGAGC
GTTGCTGGTGGAACGCGTTCGGTGAGTCGTACCGTGCGTATCGTGAAGATATGCTTAAACGCGACACTCTCGAACTATCACGCTAC
GTTGCGTCGATGGCTCGTCAAGCCGGGCTGGCTGAACTCACTCCCATTGATTTGGAGGTGCTTGCTGACCCGAACAAACTCCAGTA
TAAGTGGACCGAGGCCGATGTCTCGGCGAATATCCACGAGGTACTGATGCATGGCGTATCGGTCGAAAAGACTGAGCGCTTTCTCC
GTTCTGTAATGCCGAGGTAA

Spike protein, P3 in Segment M, (NCIB gene ID:956441)

https://www.ncbi.nlm.nih.gov/gene/?term=956441

ATGCGCTACCAAGGCATCAACGAGTGGCTGGGTGGAGCCAAGAAACTCACCACCGCAAACGGTGAGATTGGCGCTATCTACCTCT

CCGCTGCTCCTCCCACCGACGCCGCACGTGCGGACGCTAAGGCGGTGGATTTTACTGCTGGTTGGCCAAGCGCGATCGTTGACTGC
GCTGATGCCACTCGTGCCAAGCAGAACTACCTGTGGGTTGGCGATAACGTTGTGCACATCGGGGCTAAACACGTTCCACTCCTCGA
TCTGTGGGGCGGGACAGGTGATGCCTGGCAGCAGTTCGTTGGCTATGCCTGCCCAATGCTCGACCTTTGTCGTGCGTGGGGCCTGG
GTTATGCCAGCGCTTCTGTAACCACCGGCTCGTTGCAGGGCTATCAGCCATCGGCGTTCTTGGACGTTGAGCAACAGCAGTTCGCG
AAGGACAATCTCAACCTGTATGGCGATAACTGCCTTGACCTGGCCACCAGTTCGTCCGCTCAGCGGGCATTTCTGGAGCAGTGCAT
GGGCTGCGCCTTGCCGGAGGATTGCATCTTCGGTTGGTATGTGAAAATGGATTGGGAAGGTTCGGCAGTTGCCGACGCCTACGCTG
CGATCCGTGTCCAAGGGTTCGCCACTGTAATGGCACCTTGGCAGTCGGTTGGCGGTGCTGGCTACGTTTACGCTCGTGTGCCTCAA
AAAGGCGCGTGGATGGGTGTGAACCTGCTTGCCTATGTCCACGGCACCAGTGGCCAGCCTGCTTATGGCATTCCGATGACCCTCTC
GGGGTTCACCGGTAACATGGGTCAGGTGGCTTCGAAGTGGCTCATGCTTCCTCTCCTGATGATCGTCGACCCTCATGTCGTCCAGAT
TTTGGCCGCACTGGGGGTTAAACGTGGGACCAAATCGGACCCACGGACGACCGACGTGTACGCTGATCCGAAGGTTCCGGCTAGC
CGTATTTCCGGGCCGATGATCAATGGAACGGTTGCTCCTCCTGCGACGATCCCCGCTACCATTCCGGTGCCTCTGGCGCCGCTCGGT
GGCGCGGGTGGCCCTGGCGCTCAGGGTTTCCAGGTATACCCCGTTTTCACCTGGGGTCTGCCTGAGTTCATGACCGACGTGACCAT
CGAAGGTACCGTCACTGCGGACTCCAACGGTCTGCATGTCGTGGACGACGTGCGTAACTACGTCTGGAACGGTACTGCTCTTGCTG
CAATTGAGCAGGTCAATGCCGCTGACGGTCGAGTTACGCTCACTGACTCTGAGCGTGCTCAACTCGCCTCGTTGACTGTTCGAACC
GCATCGTTGCGTCAGCAGCTGTCGGTTGGGGCAGACCCCTTGTCCAAGACGTCGATCTGGCGTCGGGCTCAAAAGGCCGATTATG
ATCTGCTGTCTCAACAGATCATCGAAGCGGACACGGTGAAAAACCTACCTGCTGTGACGTTCGCTCAGGCGAACAAAGCGGCAGG
CGGTCAATCCGAGACGTTGTGGCACCAGATGTATCGGGTCAACGATATCGCTGGCGATCAAGTCACCGCAATCCAAATCACTGGTA
CGATGGCGACTGGCATTCGATGGTCGGCAACTGCTGGCGGTCTGGTCGTCGATGCTGACGAGCAAGATGCGGTGATCGCGATTTCG
TCCGGTAAGCCGGTCAAGAACAGCTCCGACCTTCCTACGGCCGACGCTGTGAACTACTTGTTCGGTATCACTGCGGACGATATGCC
TGGTATCGTTTCCTCGCAAAAGGAAATGAACAGCGAGTTTGAAGAAGGTTTCCTTCAGAAAGCTCGTCTCTGGAACCCACGTAAG
CTCGTCGAAAACGTCCAGAATGCCTATTTCCTGATGGTGTACGCTCGCGATCGGAAGCAATTCCACTCGTTGGTGGCATCCTCTCTG
GCGATGGCCAAGCTGGGCGTAAGTACGCGGGCCTGTAAGGAGTCGTATGGCTGCTGA

Morphogenetic protein, P12 in Segment S, (NCIB gene ID:956431)
https://www.ncbi.nlm.nih.gov/gene/?term=956431
ATGGTTATCGGTCTCCTGAAGTATCTCACGCCTGCCGTTAAGGTGCAGATGGCTGCTCGCGCGTTGGGCCTGTCCCCCGCCGAAGT
CGCTGCAATTGACGGCACGTTGGGTCGTGTCTCTGCGATGCCAGCGGTCGCGGTCGTGCTGGGAGGGAAACCTCTCTCTCTGGCC
ACGATCGCGTCAGTTGTGTCTGATGCAAACCCCAGTGCCACTGTTGGCGCGCTTATGCCTGCTGTACAGGGCATGGTGAGTTCCGA
CGAAGGCGCGAGTGCGTTGGCTAAGACCGTGGTAGGCTTCATGGAGTCCGACCCCAACAGCGATGTCCTGGTTCAACTGCTCCAC
AAGGTGTCAAACTTGCCGATTGTCGGCTTTGGTGACACGCAGTATGCAGACCCAGCTGACTTCTTGGCCAAGGGAGTTTTCCCTCT
GATCAGGAAGCCAGAAGTAGAGGTTCAAGCTGCGCCTTTCACCTGTCGTCAGTGTGATCATGTTGATCACATCACTGATGTACCTC
AAACTTCGACCTTTGTTCACAAATGCACTTCGTGCGGCTTTGTGCAGATGGTCCACCGTAAGGATGTTCCGTAA

FIG S2. Characterization of vacuoles from *S. cerevisiae*

(a)

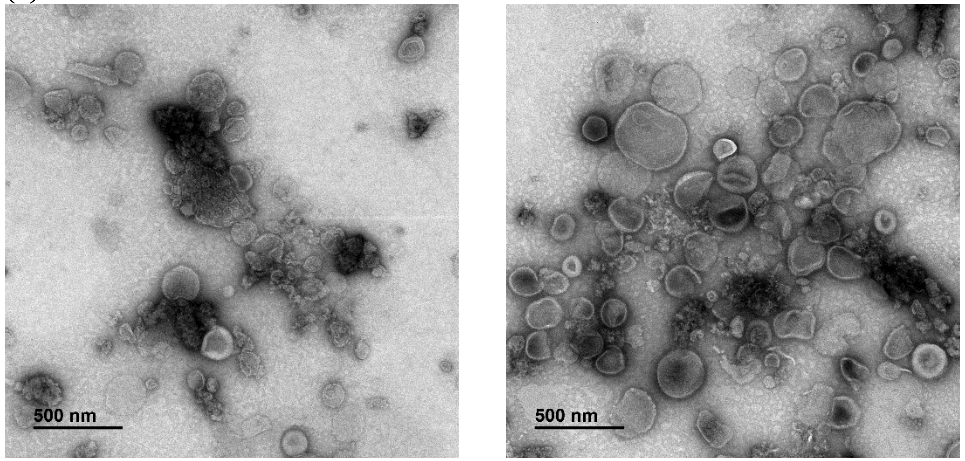

(b)

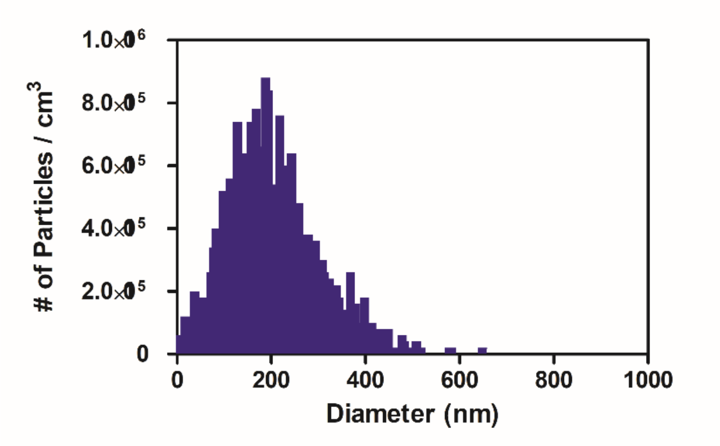

Characterization of vacuole from *saccharomyces cerevisiae* using (a) TEM and (b) nano particle tracking analysis.
